# Integrated Single-Strand DNA Capture and Haplotype-Guided Variant Calling Platform for Non-Invasive Prenatal Testing of Monogenic Disorders

**DOI:** 10.3390/diagnostics16152344

**Published:** 2026-07-27

**Authors:** Jie Shen, Wei Liu, Li Lu, Li Yu, Yin Wang, Ningyuan Zhang, Fei Lin, Zhenyu Diao, Huijun Li, Rui Xiao, Xiangyu Zhu, Jun Zhang

**Affiliations:** 1 Department of Clinical Laboratory, Sir Run Run Shaw Hospital, School of Medicine, Zhejiang University, Hangzhou 310016, China; 21718578@zju.edu.cn; 2 National Engineering Laboratory for Key Technology of Birth Defect Control and Prevention, Screening and Diagnostic R and D Center, Hangzhou 310020, China; 3 Biosan Clinical Laboratory, Hangzhou 310020, China; 4 Center for Obstetrics and Gynecology, Nanjing Drum Tower Hospital, The Affiliated Hospital of Medical School, Nanjing University, Nanjing 210008, China; 13851620552@163.com (W.L.);; 5 Center for Reproductive Medicine and Obstetrics and Gynecology, Nanjing Drum Tower Hospital, Affiliated Hospital of Medical School, Nanjing University, Nanjing 210008, China; 6 Center for Molecular Reproductive Medicine, Nanjing University, Nanjing 210008, China

**Keywords:** non-invasive prenatal testing, monogenic disorders, single-strand capture targeted sequencing

## Abstract

**Background/Objectives**: Non-invasive prenatal testing for monogenic disorders (NIPT-MD) provides a safe alternative to invasive procedures. However, its clinical utility is often limited by challenges such as low fetal fractions (FF), technical artifacts, and the difficulty in resolving maternally inherited pathogenic variants from the overwhelming background of maternal cell-free DNA (cfDNA). This study aimed to develop and validate a robust NIPT-MD platform for monogenic disorders, applicable across major common Mendelian inheritance patterns, by enhancing the accuracy of fetal variant detection in cfDNA. **Methods**: We developed a novel NIPT-MD platform validated using both simulated samples and a retrospective clinical cohort. The workflow involves a single-strand capture library preparation incorporating unique molecular identifiers (UMIs) to mitigate amplification artifacts. FF was precisely quantified using a fixed panel of high-minor-allele-frequency single-nucleotide polymorphisms (SNPs). Fetal genotypes were then inferred by resolving parental haplotypes through a statistical model that integrates weighting of variant allele frequency (VAF) and haplotype-informative SNP counts. **Results**: The single-strand capture protocol incorporating UMIs significantly reduced amplification biases. Validation of the fetal DNA fraction estimation algorithm revealed strong concordance with a Y-chromosome-derived method and expected spike-in samples. Optimization of the FF estimation panel conferred greater experimental stability. The platform reliably detected both paternally and maternally inherited pathogenic variants. In a retrospective cohort of 35 clinical cases, the NIPT-MD platform achieved high concordance with genotypes determined by invasive testing. **Conclusions**: These findings present an accurate and robust NIPT-MD platform for monogenic disorders, with high concordance to invasive testing validated in a retrospective cohort of 35 clinical cases. This method holds promise for clinical application in managing families at high risk of monogenic diseases.

## 1. Introduction

Monogenic disorders, encompassing autosomal dominant (AD), autosomal recessive (AR), and X-linked inheritance patterns, account for a substantial proportion of inherited diseases globally. These conditions, caused by pathogenic variants at single genetic loci, frequently manifest as severe phenotypes that result in significant morbidity and lifelong health challenges. Epidemiological analyses estimate the combined incidence of monogenic disorders to be approximately 1 in every 100 live births worldwide, underscoring their considerable public health impact [1]. In a screened cohort of 16,669 Chinese families receiving expanded carrier screening, the combined carrier frequency was 55.58% for 197 autosomal genes and 1.84% for 26 X-linked genes; critically, 5.24% of couples identified as at risk for severe monogenic disorders [2]. Traditionally, prenatal diagnosis has relied on invasive procedures (e.g., amniocentesis), which carry inherent risks including miscarriage and maternal–fetal infection [3].

Since the 1990s, the landmark discovery of cell-free fetal DNA (cffDNA) in maternal plasma has facilitated non-invasive prenatal testing (NIPT) development [4,5,6], particularly for common fetal chromosomal aneuploidies (e.g., trisomy 21, 18, and 13) [5,7]. Building on this success, non-invasive prenatal diagnosis (NIPD) aims to extend this non-invasive strategy to the precise detection of monogenic disorders. Recent advances in high-throughput sequencing, particularly targeted ultra-deep sequencing [8,9,10,11], digital polymerase chain reaction (dPCR) [12,13,14], and advanced bioinformatic algorithms, such as the sequential probability ratio test [5,15] and the Bayesian algorithm [16], have led to significant improvements in the detection of single-gene disorders. However, notable limitations persist, particularly involving complex inheritance patterns, low fetal fractions (FF), or challenging familial genotypes.

While NIPD initially proved feasible for detecting paternally inherited or de novo variants [17,18,19], the identification of maternally inherited pathogenic alleles remains a major technical obstacle due to the overwhelming background of maternal cfDNA [20,21]. Furthermore, many existing methods of NIPT for monogenic disorders experience reduced accuracy at fetal fractions below 4–5%. Prior studies also often focus on a limited set of disorders or specific inheritance patterns (e.g., paternal autosomal dominant), limiting broader clinical utility. Consequently, currently available gene panels may not meet the specific diagnostic needs of high-risk families and lack applicability across diverse inheritance patterns.

To address these challenges, we have developed an integrated proof-of-principle platform combining a single-strand DNA capture and extension system with next-generation sequencing (NGS). We hypothesized that single-strand capture would improve the recovery of highly fragmented cfDNA compared to conventional double-stranded PCR. Unique molecular identifiers (UMIs) are employed to facilitate precise molecular counting and suppress PCR and sequencing artifacts. In addition, targeted high-throughput sequencing of selected single-nucleotide polymorphism (SNPs), coupled with haplotype analysis and a calculated support score (H score), facilitates quantitative assessment of allelic imbalances and accurate inference of the fetal genotype across various inheritance patterns.

This study aimed to develop a non-invasive prenatal testing platform for monogenic disorders (NIPT-MD) that integrates single-strand DNA capture with haplotype-guided analysis, and to systematically evaluate its performance across major Mendelian inheritance patterns using both simulated samples and retrospective clinical samples. This platform will deliver reliable detection performance and provide a clinically accessible, practical testing option for families with specific clinical needs or high genetic risk.

## 2. Methods

### 2.1. Study Design

The study design consisted of two phases: (1) a proof-of-concept phase to evaluate the feasibility of the fetal fraction estimation method using simulated fetal DNA fragments, which were generated through the ultrasonication-mediated fragmentation of offspring genomic DNA (gDNA) mixed with maternal gDNA; (2) an exploratory clinical evaluation phase to assess the analytical sensitivity and specificity of the customized, family-specific approach using 35 independent plasma sample cohorts containing predefined pathogenic variants. All experiments and bioinformatic analyses were performed in a blinded manner, with personnel masked to fetal genotypes from invasive diagnostic procedures until all NIPT-MD results were finalized.

### 2.2. Sample Collection and Preparation

This study was approved by the Medical Ethics Committee of Nanjing Drum Tower Hospital affiliated with Nanjing University Medical School in accordance with the Declaration of Helsinki (Approval No. 2022-527-02). Pregnant women (*n* = 35) referred for prenatal diagnosis with a history of offspring affected by monogenic disorders underwent trio-based genetic testing, which identified the known pathogenic variants between 7 weeks and 24 weeks of gestation, and were enrolled after providing written informed consent. For fetal genotype analysis, peripheral blood samples from the parents and the affected fetal sibling (proband) were collected. For orthogonal experimental verification, amniotic cells or chorionic villus sampling (CVS) samples were collected and analyzed via custom Sanger sequencing.

For families with prior whole-exome sequencing (WES) data from the proband, the NIPT-MD analysis can be performed directly on maternal plasma cfDNA; otherwise, peripheral blood samples from the mother, father, and proband (or other informative family members) are required for genomic DNA extraction and haplotype phasing. Genomic DNA from peripheral blood (200 μL) was isolated using the TIANamp Genomic DNA Kit (TIANGEN, Beijing, China). CfDNA was extracted from 2 mL maternal plasma using the Magnetic Serum/Plasma DNA Maxi Kit (TIANGEN, China) and eluted in 40 μL of buffer. Amniotic fluid samples were centrifuged (3000× *g*, 10 min, 4 °C) to pellet cellular components; following supernatant removal, the pellets were processed with the TIANamp Micro DNA Kit (TIANGEN, China) according to manufacturer protocols. DNA concentrations were quantified using the Qubit 3.0 Fluorometer with the dsDNA HS Assay Kit (Yeseen, Shanghai, China).

### 2.3. Probe Design

For the estimation of the fetal DNA fraction, a fixed panel of 322 SNP loci (MAF of 0.2–0.8, GC% of 40–60%, no homology) spanning 22 pairs of autosomes and sex chromosomes was initially selected. Through an evaluation of variant allele frequency (VAF) fluctuations, locus density, and background noise levels, 132 high-confidence SNP loci meeting a 2% VAF cutoff threshold were retained as the reference panel. For genotype analysis, in trios with WES, informative SNPs within 10 Mb of target variants were used for primer design. In families with known haplotypes, ≥14 informative SNPs (Maternal ≥ 9, Paternal ≥ 5) flanking the pathogenic variant were selected for primer design. Primer3 software was utilized to generate 25–35 bp primers positioned ≥10 bp from SNP sites, optimized for annealing temperatures between 60 °C and 70 °C. All primers underwent in silico specificity validation via BLASTN alignment against the hg19 reference genome to exclude secondary structure formation and non-specific binding.

### 2.4. Library Construction and Sequencing

Extracted cfDNA fragments underwent end-repair and A-tailing. To simulate cfDNA for the proof-of-concept phase, gDNA samples (50–100 ng) were mechanically fragmented to 150–200 bp prior to end-repair. Both nucleic acid types were subsequently ligated with UMI-containing adapters. Targeted enrichment was achieved through 8-cycle PCR amplification with indexing primers, followed by final library amplification. Library molar concentrations were quantified using the ABI 7500 Real-Time PCR System (Applied Biosystems, Foster City, CA, USA). Pooled libraries were denatured and sequenced on an NGS platform (paired-end 150 bp mode).

### 2.5. Sequence Data Processing

Raw sequencing data underwent quality control processing using *fastp* software v0.23.0, including adapter trimming, the removal of reads shorter than 50 bp, poly-G sequence elimination, and UMI sequence extraction with flanking sequence removal. Processed reads were aligned to the hg19 reference genome via Sentieon’s BWA-MEM algorithm, followed by coordinate sorting with Samtools. UMI-based deduplication and local realignment were performed using UMI-tools and Sentieon’s Realigner, respectively. UMI deduplication utilized a directional deduplication method, an edit distance threshold of 1, an underscore as the UMI separator, and paired-end mode. Variants were called using Basecounts software v0.1.0 and outputted in a standardized VCF format.

### 2.6. Fetal DNA Fraction Estimation

Fetal DNA fraction was estimated using the reference panel of 132 high-frequency SNPs. VAFs of these SNPs were quantified using Basecounts software for fetal DNA fraction estimation. A noise threshold was systematically applied to eliminate background stochasticity. The FF model estimates fetal DNA fraction through a locus-specific framework based on genotype configurations. Loci exhibiting a VAF between 1.25 and 15% or between 85 and 98.75% correspond to regions where the mother is homozygous and the father is heterozygous. These loci are classified as fetal variants markers and are utilized for estimating fetal fraction. For loci within the VAF range of 1.25–15% (reflecting fetal-inherited alternative alleles), the locus-specific fetal fraction is calculated as *F*_site_ = 2 × VAF_site_. Conversely, for loci in the range of 85–98.75% (representing fetal-inherited reference alleles), the fetal fraction is determined using *F*_site_ = 2 × (1 − VAF_site_). The global FF is subsequently derived by averaging all valid locus-specific estimates (*F*=$\frac{1}{N}$ $\Sigma_{\mathrm{i}=1}^{N}Fsite\left(i\right)$), where *N* denotes the total number of qualified markers.

### 2.7. Haplotype Phasing Using Family-Based WES Data

Haplotype phasing was performed using a standard pedigree-based analysis pipeline. Raw sequencing reads were processed through quality control, adapter trimming, and aligned to the GRCh37 reference genome. Variant calling and joint genotyping were conducted following the GATK Best Practices workflow. Variants were filtered using standard hard-filtering criteria (QD < 2.0, FS > 60.0 for SNPs), and genotypes were evaluated for Mendelian consistency. Haplotype reconstruction was executed using the GATK PhaseByTransmission tool on filtered variants, leveraging parent–offspring relationships to resolve allele transmission. Phasing accuracy was verified by confirming a low Mendelian inconsistency rate. The final output provided phased genotypes in VCF format for downstream analyses.

### 2.8. NIPT-MD Analysis of Parental Transmitted Variants

Initial quality control excluded SNP loci with a sequencing depth below 500× to ensure sufficient coverage (targeting a minimum of 5–10 fetal reads per allele) for reliable detection. Diagnostic loci are defined by parental genotype discordance: paternal-informative sites occur where one parent is homozygous and the other heterozygous, while maternal-informative sites exhibit the complementary genotype configuration. The familial haplotype carrying the known pathogenic variant was consistently designated as Haplotype 1 (H1), with the other allele assigned as Haplotype 2 (H2).

For paternal inheritance analysis, two SNP categories were defined: father_H1 (alleles in paternal H1 matching maternal genotypes) and father_H2 (alleles in paternal H2 matching maternal genotypes). Fetal inheritance of paternal H1 was inferred if the VAF of father_H1 approached 1.0, while father_H2 VAF ≈1 − *F*/2 (where *F* = fetal DNA fraction). Conversely, paternal H2 inheritance was indicated by father_H2 VAF ≈1.0 and father_H1 VAF ≈1 − *F*/2. Maternal inheritance haplotype analysis utilized maternal_H1 (maternal H1 alleles matching paternal genotypes) and maternal_H2 (maternal H2 alleles matching paternal genotypes). Inheritance of maternal H1 yielded maternal_H1 VAF ≈0.5 + *F*/2 with maternal_H2 at baseline, whereas maternal haplotype 2 inheritance reversed this VAF deviation.

To quantitatively determine the fetal haplotype origin, a haplotype support score (H score) was formalized for each SNP. The H-score represents a weighted value of the deviation between the observed VAF and the theoretical VAF expected based on the estimated fetal fraction. For each informative SNP, the algorithm calculates a score based on the deviation of its observed VAF in cfDNA from the theoretically expected VAF under a given inheritance hypothesis. The score is calculated as H-score = max(0, S_max_ − P×|VAF_obs_ − VAF_exp_|), where S_max_ is the maximum possible score for a single SNP, set to 100. P is a penalty coefficient (set to 750) that penalizes deviations. VAF_obs_ is the observed VAF of the SNP allele after UMI-based error correction. VAF_exp_ is the theoretically expected VAF, which depends on the FF and the inheritance scenario (e.g., for an autosomal locus where the fetus inherits the informative allele, VAF_exp_ = 0.5 + *F*/2; if not, the VAF of that allele is 0.5 − *F*/2). Each informative SNP was assigned to the haplotype with the highest H score. The total number of SNPs supporting H1 and H2 was then counted. A haplotype was designated as inherited if it met two criteria: (1) it was supported by a greater number of SNPs, and (2) the H score of its supporting SNPs was higher than that of the alternative haplotype. Instances failing to satisfy both criteria, or exhibiting ambiguous VAF deviations, were systematically classified and reported as non-conclusive (NC).

### 2.9. Statistical Analysis

Statistical analyses were performed using GraphPad Prism version 8.0 (GraphPad Software, San Diego, CA, USA). Group comparisons were analyzed using an unpaired Student’s *t*-test. Linear regression analysis was applied to scatter plots to compare the FF estimation; the coefficient of determination (R^2^) and 95% confidence intervals (95% CI) were reported. Statistical significance was defined as *p* < 0.05.

## 3. Results

### 3.1. Development and Validation of Single-Strand Extension Technology and Fetal Fraction Estimation Model

**Figure 1:**
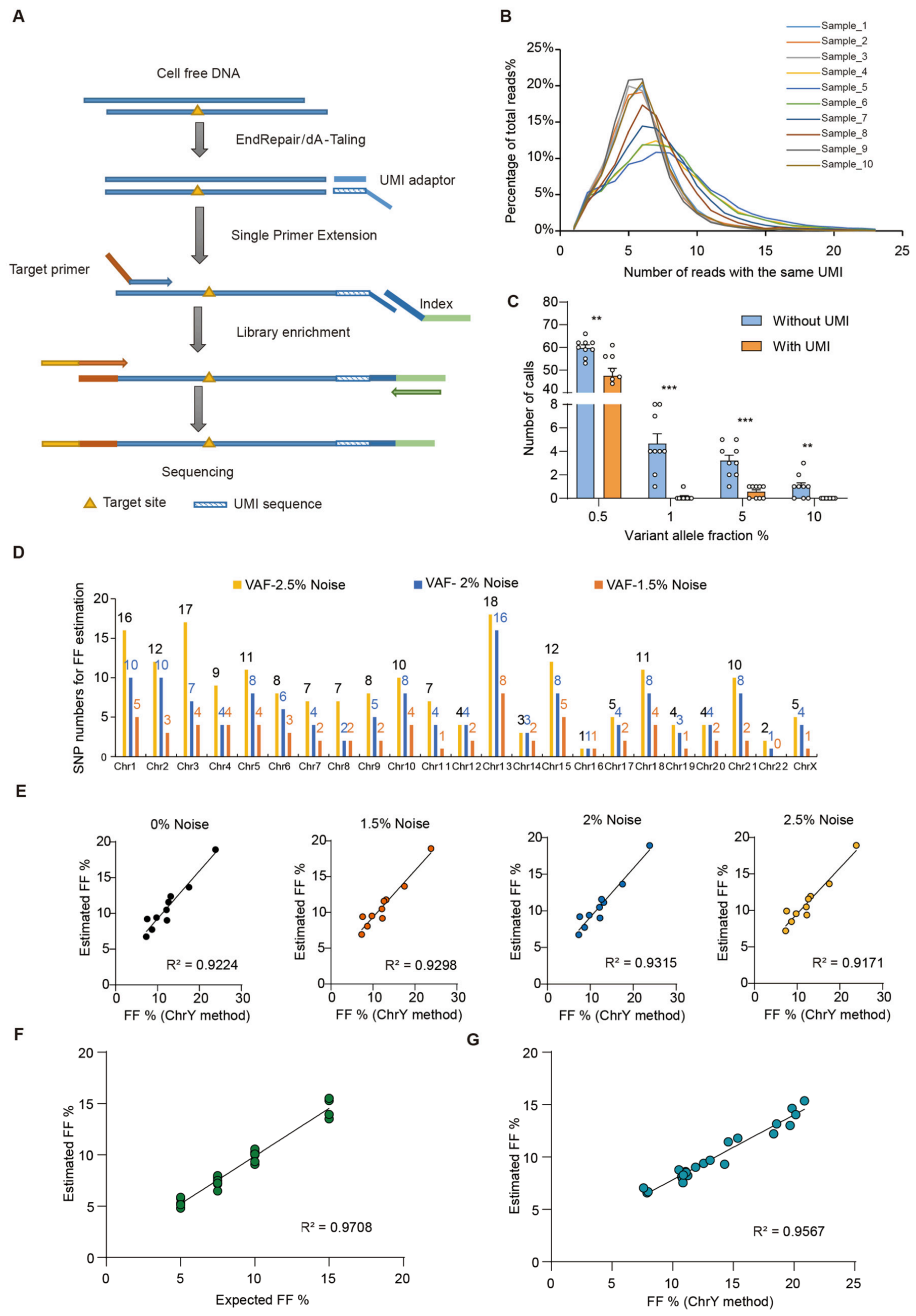
Schematic of NIPT-MD workflow and validation of fetal fraction estimation model. (**A**) Workflow for NIPT-MD library preparation. DNA samples after end-repair and A-tailing were ligated to UMI adapters (8 bp). Target-specific amplification was achieved through targeted single-chain primer extension followed by PCR amplification with indexed primers (8 bp). Enriched libraries were sequenced on an NGS platform. (**B**) UMI redundancy analysis. Read counts sharing identical UMIs are shown for 10 representative samples. (**C**) Comparative distribution of SNP variant calls with versus without UMI correction (*n* = 9). Data are stratified by VAF intervals: 0–0.5%, 0.5–1%, 1–5%, and 5–10%. Values are represented as mean ± SEM. ** *p* < 0.01, *** *p* < 0.001 (Student’s *t*-test); n.s., not significant. (**D**) Chromosomal distribution of FF-informative SNPs filtered by distinct VAF noise thresholds (1.5%, 2%, 2.5%). (**E**) Correlation between FF estimated by the SNP panel-based algorithm and Y-chromosome-derived NIPT methodologies under varying noise conditions (0%, 1.5%, 2%, 2.5%). (**F**) Correlation between FF estimated by the SNP panel-based algorithm and expected FF values in spike-in samples simulating 7 independent families. (**G**) Correlation between FF estimated by the SNP panel-based algorithm and Y-chromosome-derived NIPT methodologies in 23 pregnancies. Pearson correlation coefficient (R) and regression line are shown.

To optimize template utilization efficiency, single-strand DNA probes were designed to capture target DNA fragments, followed by ligation with universal adapters containing an 8 bp UMI. As illustrated in Figure 1A, cfDNA from maternal plasma or family gDNA from peripheral blood undergoes single-strand extension utilizing primers targeting specific SNP loci. The integration of randomized UMI sequences during library construction effectively mitigated noise resulting from sequencing artifacts and PCR amplification biases. To evaluate the performance of UMI-based error correction, reads sharing identical UMI sequences were collapsed into consensus sequences (Figure 1B). This bioinformatics filtering significantly diminished background noise, particularly in samples with a VAF of ≤10% (Figure 1C).

For FF estimation, a customized panel comprising 132 SNPs with high minor-allele-frequencies, distributed across autosomal and sex chromosomes was utilized (Figure 1D and Supplementary Information Table S1). To mitigate biases introduced by technical variance in VAF measurement, customized SNPs for FF estimation were evaluated using incremental thresholds of 0%, 1.5%, 2% and 2.5% of VAF noise compared with Y-chromosome-based FF estimates (Figure 1E). Based on the accuracy evaluation of FF estimation across these distinct thresholds, a 2% global noise threshold was ultimately applied to the final allele frequency analysis. This threshold provided an optimal balance between effectively reducing technical artifacts and retaining a sufficient number of informative SNPs to ensure robust FF estimation (Figure 1D). To validate the SNP-based FF estimation model, the estimated FFs were plotted against expected concentrations in spike-in DNA controls and conventional Y-chromosome-derived NIPT methodologies. The SNP panel-based algorithm exhibited minimal deviation from expected theoretical concentrations (R^2^ = 0.9708; Figure 1F) and maintained high concordance with Y-chromosome-based FF estimates (R^2^ = 0.9567; Figure 1G). These findings confirm that the single-strand capture extension methodology, combined with UMI integration and a stable SNP panel for FF estimation, facilitates precise determination of SNP allelic fractions, thereby minimizing downstream errors in NIPT-MD analysis.

### 3.2. Analytical Validation Using Expected Spike-In Family Models

**Figure 2:**
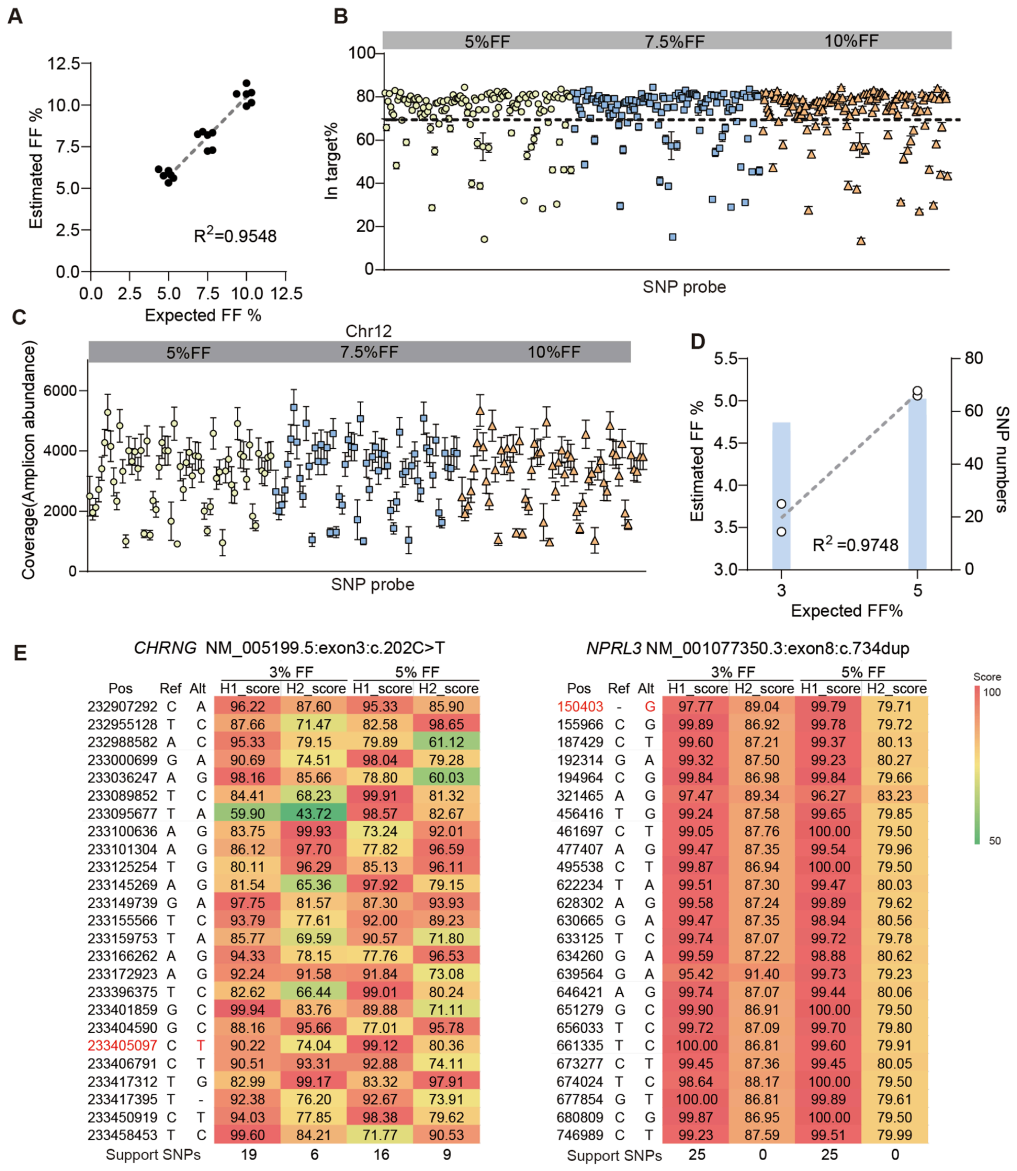
Analytical validation of the NIPT-MD platform utilizing expected spike-in family samples. (**A**) Correlation between the FF estimated by the SNP panel-based algorithm and expected FF in spiked-in samples simulating 6 independent families (FamilyA–FamilyF). (**B**) In-target capture efficiency of 132 core SNP loci across simulated fetal DNA fractions (5%, 7.5%, 10%). Data represent mean ± SEM for biological replicates (*n* = 6). (**C**) Sequencing depth uniformity across 61 informative SNPs on chromosome 12, analyzed in 6 familial spike-in models. (**D**) Estimated FF plotted against FF of expected spike-in family samples. (**E**) Validation of genotype detection accuracy using simulated family samples with low FFs (3% and 5%). Heatmaps are shown for maternally inherited pathogenic variant or informative SNPs (**left**) and a paternally inherited pathogenic variant or informative SNPs (**right**). The heatmaps visualize the haplotype support patterns across the target genomic region.

To validate the analytical performance of the NIPT-MD workflow, maternal and proband gDNA were sheared via sonication to mimic the fragmentation profile of cfDNA and subsequently spiked into mixtures at defined fractions (5%, 7.5%, and 10%). To evaluate assay robustness, a conserved genomic region (Chr12:102276987–104212582) was selected for family-specific primer design and tested across six simulated independent families. The FF estimated by the proposed algorithm showed strong correlation with theoretical spike-in DNA concentrations (R^2^ = 0.9548; Figure 2A). Capture efficiency was determined by evaluating the in-target rates of the 132-core SNP panel, which demonstrated a mean in-target rate exceeding 70% across all simulated mixtures (Figure 2B). A minimum sequencing depth of 1000× was achieved across target SNP sites on chromosome 12 (Figure 2C). The accuracy of the workflow was validated by comparing the NIPT-MD-predicted fetal haplotypes and genotypes with the proband’s genotypes obtained from WES. Concordance analysis across the six independent families confirmed the high accuracy of both haplotype phasing and genotype prediction (Supplementary Information Figure S1A and Table S2). Furthermore, assay stability was confirmed through three independent technical replicates of two simulated independent families across different FF gradients. The observed SNP-VAF showed high consistency across all three replicates, confirming the robustness and reproducibility of the proposed methodology (Supplementary Information Figure S1B,C).

For fetal genotyping, informative SNP sites where parental genotypes were complementary (i.e., heterozygous in one parent and homozygous in the other) were utilized. Family-specific primers were designed to target these loci based on the parental genotype information. To quantify the reliability of genotype prediction at each SNP, a confidence score (H-score) was calculated based on the weighted differential between the observed and the expected theoretical VAFs. These confidence scores were then used to tally the number of haplotype-informative loci supporting each predicted fetal genotype.

To benchmark the method’s sensitivity, we used simulated samples with known positive WES results, focusing on low fetal fractions of 3% and 5%—levels that challenge conventional assays (Figure 2D). A maternally inherited variant (*CHRNG* NM_005199.5: exon3: c.202C>T) and a paternally inherited variant (*NPRL3* NM_001077350.3: exon8: c.734dup), both located on haplotype 1, were analyzed. By evaluating family-specific SNP sites, both genotypes and pathogenic variants (indicated in red) were accurately detected in the two families (Figure 2E). Although distinguishing maternally inherited fetal alleles from the maternal background is inherently more complex, the haplotype-based approach successfully resolved the fetal genotype in simulated cases. Collectively, these data confirm that integrated NIPT-MD approach achieves high concordance in both FF estimation and haplotype interpretation using spike-in family models, even at fractional concentrations as low as 3–5%.

### 3.3. Validation in a Cohort of 35 Clinical Samples

**Figure 3:**
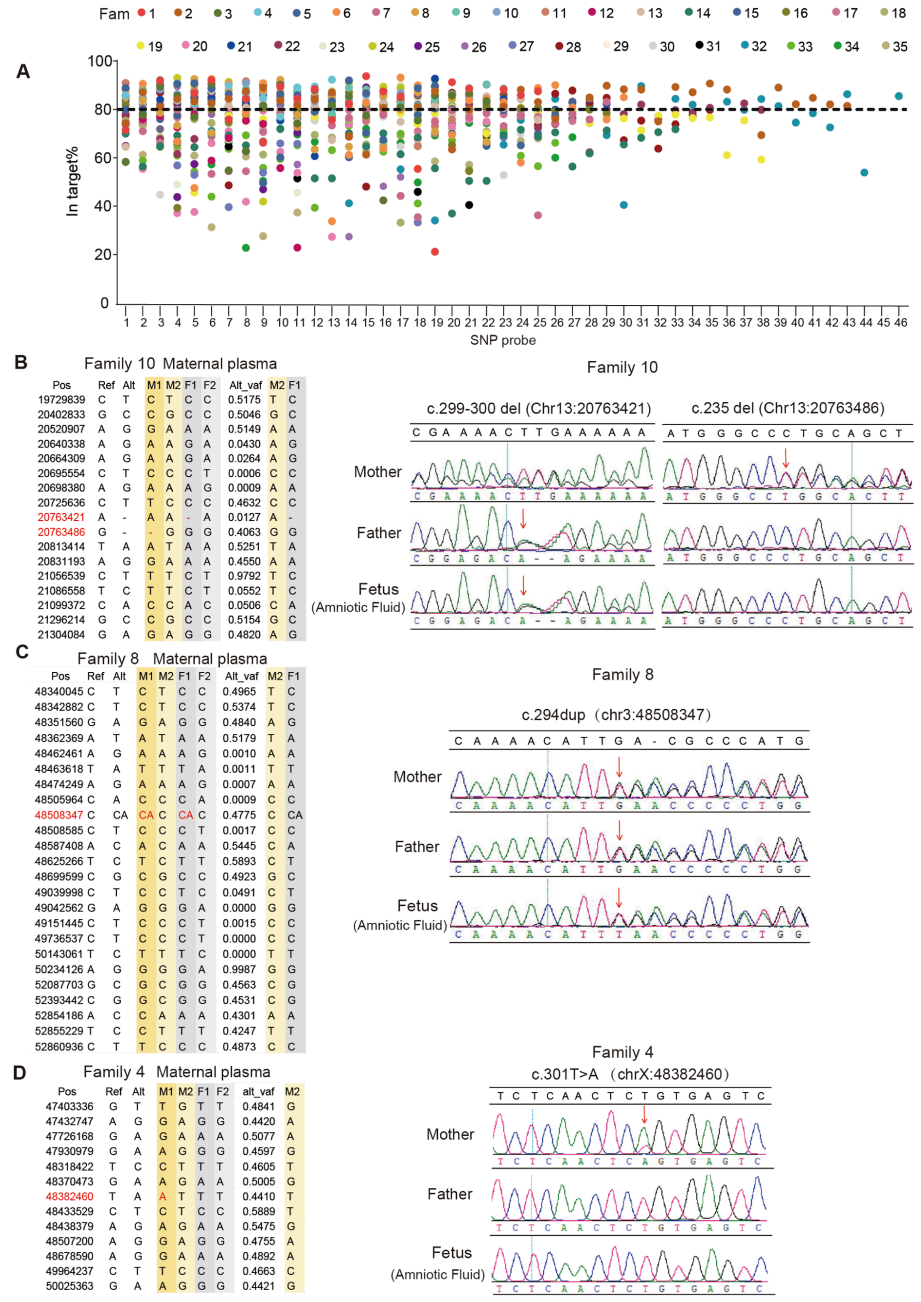
Clinical validation of haplotype resolution and diagnostic accuracy in retrospective NIPT-MD cohort. (**A**) In-target capture efficiency of informative SNPs for pathogenic region analysis across the clinical cohort (*n* = 35). Each point represents in-target rate of an individualized primer set. (**B**–**D**) Representative cases demonstrating NIPT-MD-based fetal genotype determination compared with confirmatory Sanger sequencing. Haplotypes are illustrated for three distinct inheritance scenarios: (**B**) Compound heterozygosity featuring distinct parental pathogenic variants in Family 10; (**C**) Autosomal recessive inheritance with both parents carrying an identical pathogenic variant in Family 8; (**D**) Determination of maternal X-linked inheritance in Family 4. Sanger electropherograms are shown for the father, mother, and the fetus with amniotic fluid DNA. The Red arrows indicate the position of the pathogenic variant. (Complete outcomes for the entire cohort are provided in Table 1 and Table S4).

To evaluate the clinical applicability of the NIPT-MD platform proposed herein, a retrospective cohort of 35 independent clinical samples was analyzed. Baseline characteristics are summarized in Supplementary Information Table S3. The median gestational age was 17 weeks (range: 7–24 weeks), and the median FF was 9.39% (range: 5.97–18.97%). A notable portion of the cohort (*n* = 22, 62.86%) presented with low FFs below 10%. The mutation spectrum encompassed both single nucleotide variants (SNVs; 62.86%) and small insertions/deletions (Indels ≤20 bp; 37.14%) across AR, AD, and X-linked inheritance patterns. In-target rates for each informative SNP were assessed across the 35 clinical samples (Figure 3A). The NIPT-MD assay result demonstrated high concordance with gold-standard Sanger sequencing results from matched invasive fetal samples for all 35 cases (Table 1 and Supplementary Information Table S4).

To illustrate the platform’s capability in complex scenarios, three representative cases are presented: Case 1 (Family 10—Compound Heterozygosity): The father and mother carried distinct pathogenic variants in the gap junction protein beta 2 (*GJB2*) gene (c.299-300del and c.235del, respectively). Utilizing 17 informative SNPs, the fetal haplotypes were resolved, revealing inheritance of the paternal risk haplotype (F1) and the maternal wild-type haplotype (M2), accurately predicting an unaffected carrier fetus (Figure 3B). Case 2 (Family 8—Identical Parental Pathogenic Loci): Both parents were heterozygous carriers of the same three-prime repair exonuclease 1 (*TREX1*) c.294dup variant, a scenario posing a significant analytical challenge due to dosage ambiguity. Using analysis of 23 informative SNPs, the haplotype-based algorithm determined that the fetus inherited the paternal risk haplotype (F1) and the maternal wild-type haplotype (M2), correctly diagnosing a carrier status (Figure 3C). Case 3 (Family 4—X-linked Maternal Inheritance): The mother carried an EBP cholestenol delta-isomerase c.301T>A variant. Thirteen SNPs tracked maternal haplotype 1 (M1), confirming X-linked inheritance (Figure 3D). These cases exemplify the platform’s robustness across diverse and challenging inheritance patterns. As summarized in Supplementary Information Table S5, the platform showed high concordance with invasive diagnostic results across the studied cohort. It correctly identified all 20 affected or carrier fetuses and all 15 unaffected fetuses. This corresponds to a sensitivity of 100% (95% CI: 83.89–100.00%), a specificity of 100% (95% CI: 79.61–100.00%) and an overall diagnostic concordance of 100% (95% CI: 90.11–100.00%).

## 4. Discussion

In this study, we describe the development and validation of an integrated NIPT-MD workflow that combines single-strand DNA capture, UMI-based error correction, and haplotype analysis. Our findings, based on a cohort of 35 clinical samples, demonstrate that this approach can accurately determine fetal genotypes for monogenic disorders, including those involving maternally inherited variants. This approach addresses critical technical barriers in NIPT-MD, particularly the constraints imposed by limited fetal DNA content and the analytical complexity of diverse inheritance patterns.

Current research in NIPT for monogenic disorders generally follows two paths. One focuses on specific pathogenic variants or diseases, often requiring a relatively long detection period or a limited number of SNP markers. Furthermore, the detection of maternally inherited variants is frequently inaccurate, making them difficult to distinguish. Recent advances highlight NIPD’s potential in diagnosing monogenic disorders such as β-thalassemia and congenital adrenal hyperplasia (CAH) [22]. By analyzing parental and fetal genomic DNA, SNP-based linkage analysis enables phasing of high- and low-risk alleles through relative haplotype dosage (RHDO) in Duchenne and Becker muscular dystrophies, spinal muscular atrophy and β-Thalassemia [5,23,24]. The second approach utilizes predefined gene panels for common pathogenic hotspot or high frequency regions. While useful for screening, these panels often lack the flexibility to address the specialized testing requirements of high-risk families with rare or target variants [11,17]. This study bridges these gaps by providing a versatile framework capable of identifying both paternal and maternal pathogenic variants. By leveraging parental information to phase haplotypes, the approach developed in this study enables genotype prediction regardless of the specific inheritance mode. In the retrospective cohort evaluated, the results showed high concordance with invasive testing.

Accurate FF estimation is paramount for reliable NIPT for monogenic disorders [25]. Traditionally, FF estimation has relied on sex-specific markers (e.g., Y-chromosome fragments), digital PCR or limited SNPs panel [26,27,28]. We developed and validated a fixed panel of 132 high-frequency SNPs for stable and precise FF estimation. The strong correlation of proposed SNP-based estimates with both Y-chromosome-based methods (R^2^ = 0.9567) and theoretical spike-in concentrations (R^2^ = 0.9708) underscores the reliability of this FF panel. This standardized SNP panel minimizes technical variability and provides a robust foundation for downstream fetal genotyping. A key innovation of this methodology is the use of single-strand DNA capture. Circulating cfDNA is highly fragmented and often possesses damaged ends, which can lead to the loss of informative templates during conventional double-stranded PCR library preparation, thereby reducing sensitivity [5,29,30,31]. By optimizing template utilization, the integrated single-strand capture strategy maximizes the recovery of informative cfDNA molecules. This is further enhanced by UMI-based error correction, which effectively suppresses sequencing noise and enables accurate genotype determination. The method’s performance shows some dependency on the integrity of cfDNA, with reduced efficiency for severely degraded samples.

SNP-based haplotyping features broad compatibility with diverse variant types, including single nucleotide variants, small indels and large deletions, and circumvents the technical challenge of directly calling low-frequency pathogenic variants amid a high maternal cfDNA background. However, its performance fundamentally depends on informative heterozygous SNPs in parents for accurate haplotype phasing, and may be compromised in genomic regions with low SNP heterozygosity. Moreover, meiotic recombination between the pathogenic locus and phasing-informative SNPs can lead to erroneous haplotype inference and potential misdiagnosis.

Several limitations of this study should be acknowledged. First, while the cohort of 35 samples demonstrates the platform’s clinical potential, it remains relatively small. Larger and multicenter studies are required to confirm the reproducibility of the assay across diverse populations and to better assess its performance in the rare genetic complexities like low-level mosaicism [32]. Second, the current workflow relies on prior family genomic information and known pathogenic loci, which inherently precludes the detection of de novo mutations.

## 5. Conclusions

In conclusion, this retrospective study demonstrates that our integrated NIPT-MD platform achieves high concordance with confirmatory diagnostic results across the investigated autosomal dominant, recessive, and X-linked conditions. The performance of this platform is underpinned by an approach that combines a single-strand DNA library preparation technique, enhancing the recovery of short cfDNA fragments, with UMI-based error correction and a refined bioinformatic pipeline for haplotype analysis. The use of a standardized SNP panel for FF estimation further contributes to the reproducibility of the workflow.

These findings suggest that this NIPT-MD platform holds potential as a non-invasive testing option for high-risk families with a known parental carrier status. Future work should focus on validating this method in larger cohorts and investigating performance in challenging clinical contexts to fully realize its applicability and reliability in real-world prenatal diagnosis.

## Figures and Tables

**Table 1:** Summary of clinical features, pathogenic variants, and detection results in the 35 retrospective families.

FAM	GW(w)	Chr	Pathogenic Variants	Mother Genotype	Father Genotype	Fetal Haplotype	NIPT-MD Result	Invasive Test Result
GL1	17	Chr7	*ASNS* NM_133436:c.28A>C	Het(M1)	WT	M2:F2	WT	No detectable variants
			*ASNS* NM_133436:c.1139C>A	WT	Het(F1)			
GL2	19	ChrX	*MTM1* NM_000252:c.614C>T	Het(M1)	WT	M2	WT	No detectable variant
GL3	23	Chr9	*IARS1* NM_013417.3:c.1982 G>A	WT	Het(F1)	M2:F1	c.1982G>A het pat	c.1982G>A het pat
			*IARS1* NM_013417.3: c.3587 T>C	Het(M1)	WT			
GL4	17	ChrX	*EBP* NM_006579:c.301T>A	Het(M1)	WT	M2	WT	No detectable variant
GL5	13	Chr1	*ASPM* NM_018136.5:c.8987G>A	Het(M1)	Het(F1)	M1:F1	c.8987G>A homo	c.8987G>A homo
GL6	NA	Chr7	*COL1A2* NM_000089.4:c.1197+5G>T	Het(M1)	WT	M1:F1	c.1197+5G>T het	c.1197+5G>T het
GL7	21	Chr21	*HLCS* NM_001242784:c.1544G>A	Het(M1)	WT	M1:F2	c.1544G>A mat	c.1544G>A mat
			*HLCS* NM_001242784:c.663_664del	WT	Het(F1)			
GL8	19	Chr3	*TREX1* NM_033629:c.294dup	Het(M1)	Het(F1)	M2:F1	c.294dup het pat	c.294dup het pat
GL9	16	Chr6	*ENPP1* NM_006208:c.783 C>G	WT	Het(F1)	M2:F2	WT	No detectable variants
			*ENPP1* NM_006208:c.1165-1493G>A	Het(M1)	WT			
GL10	17	Chr13	*GJB2* NM_004004:c.235 del	Het(M1)	WT	M2:F1	c.299-300 del het pat	c.299-300 del het pat
			*GJB2* NM_004004:c.299-300 del	WT	Het(F1)			
GL11	17	Chr12	*PPP1R12A* NM_002480.3: c.725G>A	WT	Het(F1)	M1:F2	WT	No detectable variant
GL12	24	ChrX	*ARSL* NM_000047.3:c.1290_1291del	Het(M1)	WT	M2	WT	No detectable variant
GL13	12	Chr9	*IARS1* NM_013417.3:c.1982 G>A	WT	Het(F1)	M1:F1	c.1982G>A het pat	c.1982G>A het pat, c.3587T>C het mat
			*IARS1* NM_013417.3: c.3587 T>C	Het(M1)	WT		c.3587T>C het mat	
GL14	17	Chr19	*RYR1* NM_000540:c.165+5G>A	Het(M1)	WT	M1:F2	165+5G>A het mat	c.165+5G>A het mat
			*RYR1* NM_000540:c.6082C>T	WT	Het(F1)			
GL15	18	Chr4	*CC2D2A* NM_001080522:c.4384T>G	Het(M1)	WT	M2:F1	c.2999A>T het pat	c.2999A>T het pat
			*CC2D2A* NM_001080522:c.2999A>T	WT	Het(F1)			
GL16	17	Chr13	*GJB2* NM_004004:c.257C>G	Het(M1)	WT	M1:F2	c.257C>G het mat	c.257C>G het mat
			*GJB2* NM_004004:c.235delC	WT	Het(F1)			
GL17	17	Chr4	*CC2D2A* NM_001378615.1:c.3347C>T	Het(M1)	WT	M2:F2	WT	No detectable variant
			*CC2D2A* NM_001378615.1: c.1472del	WT	Het(F1)			
GL18	19	Chr17	*GUCY2D* NM_000180:c.2513G>A	Het(M1)	WT	M2:F2	WT	No detectable variant
GL19	9	Chr7	*COL1A2* NM_000089.4:c.1568G>T	Het(M1)	WT	M1:F2	c.1568G>T het mat	c.1568G>T het mat
GL20	13	Chr16	*PKD1* NM_001009944:c.3611 C>A	Het(M2)	WT	M2:F2	c.3611 C>A het mat	c.3611 C>A het mat
GL21	20	ChrX	*ZFX* NM_003410.4:c.286C>T	Het(M1)	WT	M1	c.286C>T hemi mat	c.286C>T hemi mat
GL22	13	Chr17	*MYH3* NM_002470.4:c.4681C>T	Het(M1)	WT	M1:F2	c.4681C>T het mat	c.4681C>T het mat
			*MYH3* NM_002470.4:c.1582-6A>G	WT	Het(F1)			
GL23	20	ChrX	*DMD* NM_004006.3 Exon3-7del	Het(M1)	WT	M2	WT	No detectable variant
GL24	17	Chr1	*CRB1* NM_201253.3: c.4005+2T>G	Het(M1)	WT	M2:F1	c.1576C>T het pat	c.1576C>T het pat
			*CRB1* NM_201253.3: c.1576C>T	WT	Het(F1)			
GL25	17	ChrX	*L1CAM* NM_000425:c.626G>A	Het(M1)	WT	M1	c.626G>A het mat	c.626G>A het mat
GL26	7	Chr10	*PAX2* NM_003990.3:c.343C>T	WT	Het(F1)	F2	WT	No detectable variant
GL27	17	Chr10	*CWF19L1* NM_018294.6:c.1555_1557del	Het(M1)	WT	M2:F2	WT	No detectable variant
			*CWF19L1* NM_018294.6:c.1423G>T	WT	Het(F1)			
GL28	13	Chr11	*NADSYN1* NM_018161.5:c.271del	Het(M1)	WT	M2:F2	WT	No detectable variant
			*NADSYN1* NM_018161.5:c.209T>C	WT	Het(F1)			
GL29	19	Chr13	*GJB2* NM_004004.6:c.235del	Het(M1)	Het(F1)	M2:F2	WT	No detectable variant
GL30	20	Chr2	*UGT1A1* NM_000463:c.-53_-52TA	Het(M1)	WT	M2:F2	WT	No detectable variant
			*UGT1A1* NM_000463:c.211G>A	WT	Het(F1)			
GL31	18	ChrX	*RPGR* NM_001034853.2:c.2218G>T	Het(M2)	WT	M2	c.2218G>T het mat	c.2218G>T het mat
GL32	18	Chr16	*ABCC6* NM_001171.6:c.3940C>T	Het(M1)	Het(F1)	M1:F2	c.3940C>T het mat	c.3940C>T het mat
GL33	17	Chr17	*SGSH* NM_000199:c.959C>T	Het(M1)	WT	M1:F2	c.959C>T het mat	c.959C>T het mat
			*SGSH* NM_000199:c.1297C>T	WT	Het(F1)			
GL34	18	Chr6	*LAMA2* NM_000426:c.2049_2050delAG	Het(M1)	WT	M1:F2	c.2049_2050delAG het mat	c.2049_2050delAG het mat
			*LAMA2* NM_000426:c.6574-2A>G	WT	Het(F1)			
GL35	20	Chr16	*PKD1* NM_001009944.3:c.289delG	WT	Het(F1)	M2:F2	WT	No detectable variant

Abbreviations: FAM: Family; GW: Gestational weeks; Chr: Chromosome; NIPT-MD: Non-invasive prenatal diagnosis testing for monogenic disorders; Het: Heterozygous; WT: Wild type; pat: Paternal; mat: Maternal; hemi: Hemizygous; del: Deletion; M1/M2: Maternal haplotype 1/2; F1/F2: Paternal haplotype 1/2.

## Data Availability

The data that support the findings of this study are available on request from the corresponding author. The data are not publicly available due to privacy or ethical restrictions.

## Supplementary Materials

The following supporting information can be downloaded at: https://www.mdpi.com/article/10.3390/diagnostics16152344/s1, Figure S1. Validation of NIPT-MD performance for fetal haplotype and variant allele frequency determination; Table S1. Core SNP panel for fetal fraction estimation; Table S2. Overview of expected spike-in Family NIPT-MD Results; Table S3. Baseline Characteristics of the Clinical Validation Cohort (*n* = 35); Table S4. Overview of 35 retrospective family NIPT-MD Results; Table S5. Performance of NIPT-MD Compared with Invasive Diagnostic Testing Results.
