# Supplementary material for: Integrated Single-Strand DNA Capture and Haplotype-Guided Variant Calling Platform for Non-Invasive Prenatal Testing of Monogenic Disorders
Source: Diagnostics (Basel). 2026 Jul 27;16(15):2344. doi: 10.3390/diagnostics16152344 (PMC13465468; doi:10.3390/diagnostics16152344)
Supplement: Supplementary file 1 [file diagnostics-16-02344-s001.zip › diagnostics-4393537-supplementary.pdf]

Supplementary Information

Integrated Single-Strand DNA Capture and Haplotype-Guided Variant Calling Platform for Non-Invasive Prenatal Testing of Monogenic Disorders

Jie Shen[1,2,3#], Wei Liu[4, #], Li Lu[2,3], Li Yu[2,3], Yin Wang[2,3], Ningyuan Zhang[5,6], Fei Lin[5,6], Zhenyu Diao[5,6], Huijun Li[4], Rui Xiao[2,3*], Xiangyu Zhu[4,*], Jun Zhang[1,*]

[1] Department of Clinical Laboratory, Sir Run Run Shaw Hospital, School of Medicine, Zhejiang University, Hangzhou, China.

[2] National Engineering Laboratory for Key Technology of Birth Defect Control and Prevention, Screening and Diagnostic R and D Center, Hangzhou, China.

[3] Biosan Clinical laboratory, Hangzhou, China.

[4] Center for Obstetrics and Gynecology, Nanjing Drum Tower Hospital, The Affiliated Hospital of Medical School, Nanjing University, Nanjing, China.

[5] Center for Reproductive Medicine and Obstetrics and Gynecology, Nanjing Drum Tower Hospital, Affiliated Hospital of Medical School, Nanjing University, Nanjing, China.

[6] Center for Molecular Reproductive Medicine, Nanjing University, Nanjing, China.

[#] These authors contributed equally.

* Correspondence to: jameszhang2000@zju.edu.cn (J.Z.), xiangyuzhu82@sina.com (X.Z.) or xiaorui@biosan.cn (R.X.)

This PDF file includes:

- Supplementary Figure S1
- Supplementary Tables S1 to S5

Figure S1

A

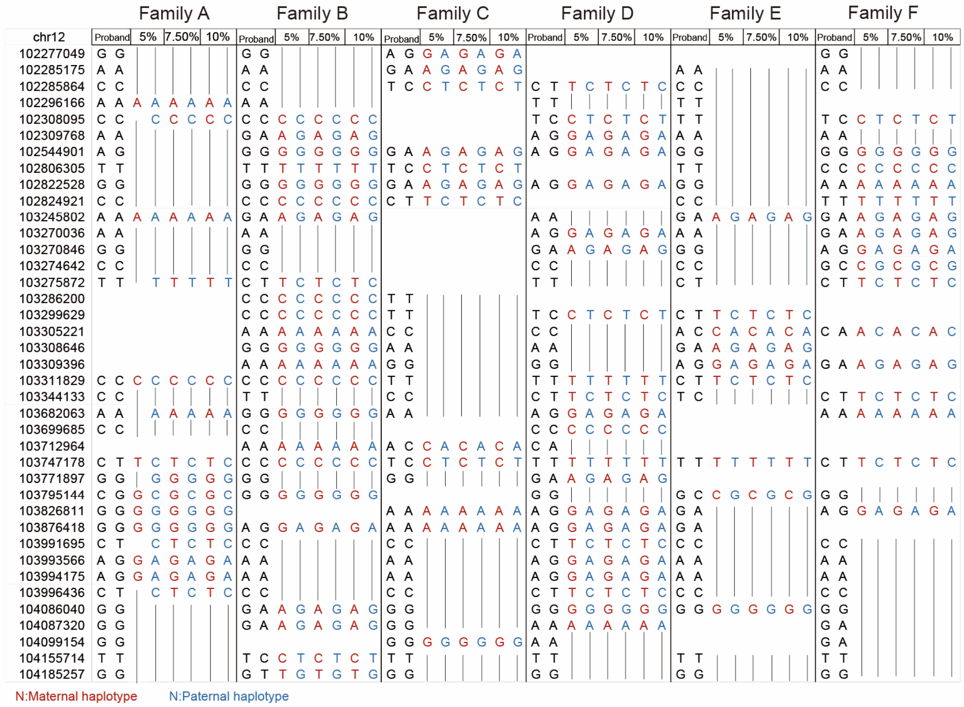

B

		5% FF			7.5% FF			10% FF		
		RUN1	RUN2	RUN3	RUN1	RUN2	RUN3	RUN1	RUN2	RUN3
chr12	102277049	0.4900	0.4985	0.5190	0.4823	0.4874	0.4790	0.4953	0.4880	0.4699
chr12	102285175	0.4890	0.5014	0.4998	0.5046	0.5497	0.5171	0.5255	0.4979	0.5208
chr12	102285864	0.5250	0.4984	0.5023	0.4777	0.4791	0.4882	0.4937	0.4870	0.4802
chr12	102288952	0.4822	0.5203	0.5032	0.4990	0.5135	0.5130	0.4984	0.5174	0.5152
chr12	102296166	0.4814	0.4763	0.4738	0.4775	0.4626	0.4557	0.4559	0.4486	0.4526
chr12	102308095	0.0293	0.0199	0.0267	0.0438	0.0418	0.0342	0.0547	0.0461	0.0411
chr12	102309768	0.5176	0.4921	0.5002	0.4924	0.5103	0.5034	0.5053	0.4956	0.5096
chr12	102544901	0.0363	0.0257	0.0255	0.0404	0.0384	0.0359	0.0584	0.0568	0.0619
chr12	102561772	0.0036	0.0081	0.0061	0.0067	0.0104	0.0106	0.0061	0.0131	0.0127
chr12	102806305	0.9674	0.9815	0.9749	0.9581	0.9620	0.9656	0.9452	0.9594	0.9473
chr12	102822528	0.9655	0.9736	0.9793	0.9627	0.9635	0.9602	0.9380	0.9547	0.9501
chr12	102824921	0.9698	0.9727	0.9776	0.9534	0.9552	0.9650	0.9409	0.9448	0.9463
chr12	103234544	0.0000	0.0007	0.0007	0.0000	0.0000	0.0000	0.0000	0.0000	0.0000
chr12	103245802	0.0005	0.0004	0.0004	0.0003	0.0004	0.0013	0.0008	0.0008	0.0006
chr12	103267770	0.0003	0.0010	0.0000	0.0011	0.0005	0.0005	0.0000	0.0000	0.0005
chr12	103269416	0.0000	0.0000	0.0004	0.0000	0.0000	0.0006	0.0000	0.0004	0.0000
chr12	103270036	0.4858	0.4917	0.5057	0.5084	0.5226	0.5157	0.4994	0.4897	0.5131
chr12	103270846	0.5166	0.5008	0.4927	0.4947	0.4903	0.4954	0.4965	0.4833	0.4976
chr12	103274642	0.0000	0.0000	0.0000	0.0000	0.0000	0.0000	0.0000	0.0000	0.0000
chr12	103275872	0.0007	0.0008	0.0006	0.0005	0.0010	0.0004	0.0012	0.0000	0.0000
chr12	103286200	0.4961	0.5102	0.4908	0.4861	0.4875	0.4832	0.5066	0.5028	0.4931
chr12	103299629	0.4966	0.5095	0.5460	0.5100	0.4856	0.5226	0.5047	0.5022	0.5222
chr12	103304171	0.5286	0.5104	0.4787	0.4998	0.5132	0.4776	0.5042	0.4893	0.5037
chr12	103305221	0.0000	0.0020	0.0005	0.0012	0.0007	0.0005	0.0003	0.0018	0.0002
chr12	103308646	0.0000	0.0009	0.0004	0.0000	0.0002	0.0000	0.0003	0.0000	0.0004
chr12	103309396	0.0008	0.0018	0.0000	0.0012	0.0011	0.0004	0.0000	0.0009	0.0023
chr12	103311829	0.0005	0.0001	0.0005	0.0005	0.0005	0.0008	0.0005	0.0004	0.0000
chr12	103312350	0.0029	0.0016	0.0014	0.0023	0.0034	0.0025	0.0013	0.0011	0.0025
chr12	103344133	0.5014	0.5086	0.5046	0.5145	0.5182	0.5008	0.5231	0.5150	0.4981
chr12	103640952	0.4439	0.4931	0.4869	0.4444	0.5027	0.4823	0.5059	0.4531	0.4769
chr12	103682063	0.4899	0.5028	0.4936	0.4779	0.4984	0.4915	0.4949	0.4908	0.4920
chr12	103699685	0.4533	0.4746	0.4648	0.4497	0.4473	0.4781	0.4561	0.4283	0.4363
chr12	103712964	0.4629	0.5238	0.4844	0.5122	0.4773	0.4871	0.5011	0.4977	0.4957
chr12	103747178	0.4670	0.4512	0.4697	0.4390	0.4670	0.4697	0.4255	0.4610	0.4538
chr12	103771897	0.4833	0.5037	0.5234	0.4933	0.4850	0.5096	0.5031	0.5149	0.4913
chr12	103795144	1.0000	1.0000	0.9995	1.0000	0.9995	0.9998	1.0000	0.9993	1.0000
chr12	103826811	0.9711	0.9769	0.9788	0.9583	0.9677	0.9634	0.9502	0.9438	0.9418
chr12	103876418	0.9693	0.9700	0.9796	0.9672	0.9570	0.9663	0.9374	0.9390	0.9466
chr12	103987895	0.4286	0.4374	0.4233	0.4147	0.4292	0.4317	0.4547	0.4502	0.4551
chr12	103988728	0.5003	0.5368	0.5217	0.5089	0.5421	0.5601	0.5302	0.5424	0.5541
chr12	103991695	0.4819	0.4892	0.4790	0.5056	0.4900	0.4954	0.4711	0.4995	0.4641
chr12	103993566	0.4977	0.4845	0.4882	0.5002	0.5071	0.4991	0.5095	0.5073	0.4874
chr12	103994175	0.5061	0.5071	0.4832	0.4970	0.4916	0.4982	0.4869	0.4839	0.5036
chr12	103996436	0.5203	0.4674	0.5008	0.4612	0.4963	0.4810	0.4768	0.4852	0.5024
chr12	104002815	0.7896	0.7479	0.8092	0.7961	0.7770	0.7757	0.8257	0.7808	0.7898
chr12	104014104	0.4695	0.4753	0.4859	0.4628	0.4983	0.4729	0.4941	0.4598	0.4927
chr12	104057868	0.4989	0.4967	0.5179	0.4873	0.4903	0.4891	0.5213	0.4942	0.4983
chr12	104059790	0.6468	0.6699	0.6455	0.6370	0.6702	0.6437	0.6712	0.6748	0.6368
chr12	104061597	0.4876	0.5295	0.4934	0.5253	0.4949	0.5239	0.5166	0.4819	0.4974
chr12	104072168	0.0000	0.0000	0.0000	0.0000	0.0000	0.0000	0.0000	0.0000	0.0000
chr12	104086040	0.5427	0.5422	0.4961	0.5735	0.5380	0.5564	0.5450	0.5739	0.5587
chr12	104087320	0.0021	0.0009	0.0014	0.0005	0.0007	0.0000	0.0011	0.0004	0.0013
chr12	104099154	0.4617	0.4908	0.4612	0.4453	0.4713	0.4603	0.4605	0.4382	0.4308
chr12	104155714	0.9993	1.0000	0.9996	1.0000	0.9996	0.9998	1.0000	0.9996	0.9996
chr12	104183431	1.0000	0.9997	1.0000	1.0000	1.0000	1.0000	1.0000	0.9991	1.0000
chr12	104183848	1.0000	0.9980	1.0000	1.0000	1.0000	1.0000	1.0000	1.0000	0.9992
chr12	104185257	1.0000	0.9996	1.0000	1.0000	1.0000	1.0000	1.0000	0.9998	1.0000
chr12	104186795	0.9983	0.9997	0.9985	0.9997	1.0000	0.9990	0.9975	0.9998	0.9990
chr12	104188476	0.9997	0.9993	0.9989	1.0000	1.0000	0.9998	1.0000	0.9997	0.9993
chr12	104196221	1.0000	1.0000	0.9993	1.0000	1.0000	1.0000	1.0000	0.9998	1.0000
chr12	104212631	0.0234	0.0265	0.0269	0.0321	0.0347	0.0378	0.0575	0.0427	0.0492

C

		5% FF			7.5% FF			10% FF		
		RUN1	RUN2	RUN3	RUN1	RUN2	RUN3	RUN1	RUN2	RUN3
chr12	102277049	0.9995	1.0000	1.0000	1.0000	1.0000	1.0000	0.9990	0.9981	0.9992
chr12	102285175	0.9991	1.0000	1.0000	0.9992	1.0000	1.0000	0.9996	0.9992	1.0000
chr12	102285864	0.9970	0.9978	1.0000	0.9979	1.0000	0.9997	0.9983	0.9978	1.0000
chr12	102288952	0.9997	0.9990	0.9991	0.9997	0.9996	0.9986	0.9997	0.9981	0.9983
chr12	102296166	0.4821	0.5120	0.4889	0.5086	0.4956	0.5150	0.4858	0.4959	0.4829
chr12	102308095	0.4971	0.5270	0.5312	0.5098	0.5162	0.5190	0.4943	0.5129	0.4992
chr12	102309768	1.0000	0.9998	0.9993	0.9996	0.9996	0.9998	0.9994	0.9985	0.9992
chr12	102544901	0.4842	0.4777	0.4687	0.4872	0.4587	0.4721	0.4546	0.4599	0.4783
chr12	102561772	0.0693	0.1650	0.0825	0.0749	0.1346	0.1034	0.1705	0.1295	0.1494
chr12	102806305	0.0008	0.0012	0.0013	0.0022	0.0004	0.0018	0.0000	0.0025	0.0000
chr12	102822528	0.0004	0.0004	0.0005	0.0004	0.0000	0.0000	0.0009	0.0004	0.0005
chr12	102824921	0.0006	0.0000	0.0000	0.0006	0.0009	0.0004	0.0006	0.0000	0.0007
chr12	103234544	0.0175	0.0227	0.0266	0.0367	0.0261	0.0321	0.0385	0.0568	0.0287
chr12	103245802	0.0256	0.0259	0.0231	0.0372	0.0263	0.0239	0.0475	0.0590	0.0494
chr12	103267770	0.0319	0.0284	0.0249	0.0423	0.0428	0.0317	0.0601	0.0578	0.0405
chr12	103269416	0.0327	0.0174	0.0213	0.0472	0.0418	0.0362	0.0583	0.0536	0.0409
chr12	103270036	0.0276	0.0345	0.0130	0.0290	0.0388	0.0450	0.0529	0.0594	0.0477
chr12	103270846	0.0291	0.0236	0.0148	0.0440	0.0402	0.0296	0.0493	0.0492	0.0424
chr12	103274642	0.0255	0.0279	0.0138	0.0312	0.0467	0.0354	0.0505	0.0537	0.0477
chr12	103275872	0.0247	0.0347	0.0181	0.0424	0.0435	0.0341	0.0498	0.0518	0.0476
chr12	103286200	0.4897	0.4900	0.4652	0.4830	0.4744	0.5155	0.4914	0.5097	0.4867
chr12	103299629	0.5127	0.5163	0.4925	0.5429	0.4577	0.5056	0.4832	0.5102	0.4922
chr12	103304171	0.4965	0.4839	0.4916	0.4949	0.4742	0.4862	0.5009	0.4883	0.4944
chr12	103305221	0.5092	0.4639	0.5137	0.4547	0.4977	0.4833	0.5006	0.5124	0.4835
chr12	103308646	0.4986	0.5241	0.4869	0.4821	0.5093	0.4921	0.5008	0.4841	0.4652
chr12	103309396	0.4959	0.5080	0.4824	0.4780	0.4973	0.5060	0.5016	0.5040	0.5040
chr12	103311829	0.4795	0.4710	0.4966	0.5169	0.5068	0.4902	0.5047	0.4970	0.5025
chr12	103312350	0.0043	0.0096	0.0029	0.0011	0.0060	0.0052	0.0041	0.0111	0.0060
chr12	103344133	0.4825	0.4899	0.5010	0.5225	0.4972	0.4866	0.5034	0.4964	0.4984
chr12	103640952	1.0000	1.0000	1.0000	1.0000	0.9991	0.9985	1.0000	1.0000	1.0000
chr12	103682063	1.0000	1.0000	1.0000	0.9992	1.0000	1.0000	0.9994	0.9995	0.9993
chr12	103699685	0.5148	0.5172	0.5275	0.5613	0.5514	0.5349	0.5550	0.5433	0.5404
chr12	103712964	0.4970	0.5293	0.5356	0.5299	0.5516	0.5206	0.5417	0.5520	0.5469
chr12	103747178	0.0313	0.0203	0.0300	0.0475	0.0334	0.0296	0.0560	0.0590	0.0389
chr12	103771897	0.4945	0.5021	0.5049	0.4981	0.4984	0.5028	0.4714	0.5013	0.4975
chr12	103795144	1.0000	1.0000	1.0000	1.0000	1.0000	1.0000	1.0000	1.0000	1.0000
chr12	103826811	0.9786	0.9797	0.9760	0.9646	0.9552	0.9639	0.9381	0.9454	0.9541
chr12	103876418	0.9742	0.9763	0.9730	0.9556	0.9550	0.9717	0.9485	0.9460	0.9565
chr12	103987895	0.0000	0.0000	0.0000	0.0017	0.0000	0.0000	0.0009	0.0004	0.0000
chr12	103988728	0.0000	0.0000	0.0007	0.0006	0.0006	0.0008	0.0000	0.0005	0.0000
chr12	103991695	0.0004	0.0000	0.0010	0.0000	0.0000	0.0000	0.0000	0.0007	0.0003
chr12	103993566	0.0000	0.0004	0.0000	0.0000	0.0000	0.0005	0.0007	0.0007	0.0004
chr12	103994175	0.0006	0.0000	0.0003	0.0000	0.0000	0.0000	0.0009	0.0005	0.0000
chr12	103996436	0.0006	0.0006	0.0000	0.0003	0.0006	0.0000	0.0020	0.0000	0.0000
chr12	104002815	0.0000	0.0031	0.0054	0.0154	0.0035	0.0000	0.0000	0.0013	0.0000
chr12	104014104	0.4637	0.4545	0.4545	0.3237	0.4412	0.4355	0.4349	0.4349	0.4473
chr12	104057868	0.4764	0.5151	0.5109	0.5051	0.4932	0.4924	0.5216	0.5045	0.4872
chr12	104059790	0.6992	0.6602	0.7167	0.6736	0.6872	0.6662	0.6369	0.6579	0.6477
chr12	104061597	0.5106	0.5325	0.4843	0.5011	0.4932	0.5269	0.5213	0.4940	0.5157
chr12	104072168	0.0222	0.0245	0.0134	0.0445	0.0359	0.0296	0.0472	0.0462	0.0362
chr12	104086040	0.9995	0.9995	0.9997	0.9995	1.0000	0.9988	1.0000	0.9993	1.0000
chr12	104087320	0.0227	0.0218	0.0224	0.0411	0.0331	0.0314	0.0562	0.0534	0.0500
chr12	104099154	0.0318	0.0241	0.0295	0.0318	0.0358	0.0297	0.0590	0.0546	0.0455
chr12	104155714	1.0000	1.0000	0.9997	0.9994	1.0000	0.9993	1.0000	0.9996	0.9998
chr12	104183431	1.0000	1.0000	1.0000	1.0000	1.0000	1.0000	1.0000	1.0000	1.0000
chr12	104183848	0.9989	1.0000	1.0000	1.0000	1.0000	1.0000	0.9995	0.9990	1.0000
chr12	104185257	1.0000	0.9998	1.0000	1.0000	1.0000	0.9998	1.0000	1.0000	0.9996
chr12	104186795	0.9997	0.9982	1.0000	1.0000	0.9976	0.9998	0.9981	0.9997	0.9995
chr12	104188476	0.9987	1.0000	1.0000	0.9997	0.9991	0.9996	1.0000	0.9990	0.9993
chr12	104196221	1.0000	1.0000	0.9995	1.0000	0.9997	0.9998	1.0000	1.0000	1.0000
chr12	104212631	0.0011	0.0002	0.0009	0.0005	0.0005	0.0008	0.0003	0.0008	0.0006

VAF
1
0

Figure S1 Validation of NIPT-MD performance for fetal haplotype and variant allele frequency determination. (A) Concordance analysis between NIPT-MD-derived fetal genotypes and the WES-derived genotypes of the probands across 6 independent families (Family A-Family F). Maternal (hg19; red) and paternal (blue) haplotypes are annotated. (B-C) Consistency of SNP-VAF across 3 independent technical replicates in 2 simulated independent families spanning distinct fetal fraction gradients (5%, 7.5% and 10%).

Table S1. Core SNP panel for fetal fraction estimation.

SNP ID	Chr	Start	End
BS-6	chr14	56584503	56584528
BS-7	chr1	28216027	28216054
BS-10	chr18	13542270	13542296
BS-18	chr2	127441561	127441586
BS-21	chr15	71125154	71125179
BS-22	chr21	35651690	35651715
BS-23	chr19	2816291	2816316
BS-26	chr21	28887289	28887316
BS-29	chr21	36644903	36644930
BS-32	chr6	138501346	138501371
BS-33	chr21	45174273	45174298
BS-39	chr13	41065337	41065367
BS-40	chr13	46952654	46952679
BS-42	chr18	38200550	38200573
BS-43	chr21	45702308	45702333
BS-44	chr18	75010266	75010291
BS-46	chr13	98824270	98824294
BS-47	chr18	46593599	46593624
BS-50	chr13	79569489	79569515
BS-52	chr2	154460832	154460858
BS-54	chr10	113157995	113158026
BS-60	chr18	3863616	3863641
BS-61	chr20	42473722	42473750
BS-63	chr13	53591380	53591408
BS-68	chr13	95675934	95675959
BS-71	chr18	61564345	61564377
BS-75	chr9	110751095	110751120
BS-78	chr18	5939633	5939667
BS-81	chr13	111624638	111624663
BS-82	chr13	43517353	43517376
BS-84	chr13	95014811	95014838
BS-86	chr13	50111579	50111604
BS-88	chr13	112759072	112759097
BS-91	chr21	47486900	47486926
BS-92	chr7	139718655	139718679
BS-93	chr13	22786851	22786876
BS-94	chr13	40345140	40345167
BS-95	chr18	56774260	56774289
BS-98	chr13	24855627	24855652
BS-100	chr13	57519137	57519162
BS-101	chr13	36103870	36103901

BS-107	chr21	41765125	41765150
BS-108	chr21	37092920	37092952
BS-115	chr1	40492695	40492720
BS-117	chr1	58655491	58655516
BS-118	chr1	66416258	66416294
BS-119	chr1	67314526	67314551
BS-122	chr1	95698927	95698952
BS-123	chr1	99597294	99597330
BS-125	chr1	156652271	156652296
BS-127	chr1	166451964	166451989
BS-130	chr1	238376600	238376628
BS-131	chr2	30887973	30887998
BS-133	chr2	66363785	66363807
BS-135	chr2	105995583	105995607
BS-137	chr2	138594279	138594304
BS-139	chr2	208905844	208905868
BS-140	chr2	210898674	210898699
BS-141	chr2	231868479	231868506
BS-142	chr2	236850042	236850067
BS-146	chr3	27884838	27884866
BS-148	chr3	32299516	32299541
BS-149	chr3	33419387	33419410
BS-150	chr3	34645545	34645567
BS-153	chr3	67299700	67299725
BS-161	chr3	182595780	182595805
BS-162	chr3	190281331	190281357
BS-164	chr4	37636690	37636715
BS-169	chr4	83379792	83379817
BS-171	chr4	97539049	97539076
BS-175	chr4	129721641	129721667
BS-180	chr5	3121955	3121980
BS-181	chr5	7617172	7617197
BS-184	chr5	45454391	45454416
BS-185	chr5	55747554	55747579
BS-187	chr5	73475155	73475182
BS-189	chr5	79251815	79251842
BS-192	chr5	158511933	158511958
BS-194	chr5	169338429	169338454
BS-196	chr6	12098568	12098598
BS-197	chr6	64193532	64193557
BS-200	chr6	76147256	76147279
BS-203	chr6	158430573	158430598
BS-205	chr6	167383039	167383064
BS-206	chr7	27925606	27925630

BS-208	chr7	91208960	91208985
BS-210	chr7	113751950	113751976
BS-215	chr8	82953927	82953953
BS-220	chr8	109520239	109520270
BS-224	chr9	37737249	37737274
BS-228	chr9	90830295	90830323
BS-229	chr9	90836422	90836450
BS-231	chr9	108442140	108442165
BS-234	chr10	14055411	14055443
BS-235	chr10	35870672	35870697
BS-237	chr10	64645907	64645941
BS-238	chr10	72910404	72910429
BS-239	chr10	82456276	82456299
BS-240	chr10	85977133	85977160
BS-243	chr10	115131763	115131788
BS-245	chr11	17994266	17994291
BS-248	chr11	46157022	46157048
BS-251	chr11	78828958	78828982
BS-254	chr11	123372916	123372941
BS-255	chr12	3033396	3033423
BS-256	chr12	14073523	14073552
BS-258	chr12	102616605	102616630
BS-259	chr12	112554914	112554939
BS-262	chr14	39917732	39917763
BS-265	chr14	92763140	92763165
BS-267	chr15	62777917	62777949
BS-270	chr15	73238467	73238492
BS-271	chr15	73267691	73267716
BS-273	chr15	81460981	81461017
BS-274	chr15	84046494	84046519
BS-275	chr15	84143484	84143509
BS-277	chr15	92687518	92687554
BS-278	chr16	31521971	31521996
BS-280	chr17	11515605	11515630
BS-281	chr17	34033036	34033064
BS-283	chr17	74877786	74877810
BS-284	chr17	81138704	81138729
BS-289	chr19	47221072	47221099
BS-290	chr19	52006734	52006759
BS-291	chr20	37635649	37635677
BS-292	chr20	40769212	40769237
BS-294	chr20	57240782	57240805
BS-296	chr22	26742792	26742814
BS-298	chrX	8895169	8895194

BS-302	chrX	47155053	47155078
BS-306	chrX	132402212	132402238
BS-307	chrX	144196816	144196841

Table S2. Overview of expected spike-in Family NIPT-MD Results

FAM	Theoretical FF %	Actual FF %	Reads	SNPs used for FF%	Mat Hap	Pat Hap	Informative SNPs used
Family A	5%	5.33%	41448018	64	NC	F1	16
	7.50%	7.24%	39484478	71	M1	F1	16
	10%	10.14%	39548688	73	M1	F1	16
Family B	5%	6.05%	32937020	64	M1	F1	23
	7.50%	8.20%	35607608	72	M1	F1	23
	10%	10.74%	35792236	75	M1	F1	23
Family C	5%	5.62%	35702166	49	M1	F1	12
	7.50%	7.29%	39003370	60	M1	F1	12
	10%	10.65%	40307688	62	M1	F1	12
Family D	5%	5.80%	13204194	56	M1	F1	23
	7.50%	8.24%	12440466	67	M1	F1	23
	10%	10.66%	12038028	68	M1	F1	23
Family E	5%	5.76%	13281328	70	M1	F1	12
	7.50%	8.33%	13344600	72	M1	F1	12
	10%	9.93%	13823276	67	M1	F1	12
Family F	5%	6.14%	14257644	49	M1	F1	20
	7.50%	8.40%	14942822	65	M1	F1	20
	10%	11.31%	13746468	63	M1	F1	20

Abbreviations: FAM: Family number; FF: Fetal fraction; Mat Hap: Maternal haplotype; Pat Hap: Paternal haplotype.

Table S3. Baseline Characteristics of the Clinical Validation Cohort (n=35)

Parameter	Total Cohort (n=35)	AR (n=21)	AD (n=7)	X-Linked (n=7)
Gestational age, weeks, median (IQR)	17 (7 – 24)	17 (12 – 23)	15 (7 – 20)	19 (17 – 24)
Fetal fraction, %, median (IQR)	9.39 (5.97–18.97)	9.39 (6.17–18.84)	10.00 (5.97–18.97)	8.60 (5.07–15.39)
Low fetal fraction (<10%), n (%)	22 (62.86%)	15 (42.86%)	3 (8.57%)	4 (11.43%)
SNVs, n (%)	22 (62.86%)	11 (31.43%)	6 (17.14%)	5 (14.29%)
Small Indel (Indels, ≤20bp), n (%)	13 (37.14%)	10 (28.57%)	1 (2.86%)	2 (5.71%)

Abbreviations: AR: Autosomal recessive; AD: Autosomal dominant; IQR: Interquartile range; SNV: Single nucleotide variant; Indel: Insertion/Deletion.

Table S4. Overview of 35 retrospective family NIPT-MD Results

FAM	FF%	SNPs used for FF%	Informative SNPs used	M1 SNPs	M2 SNPs	F1 SNPs	F2 SNPs	Mat Hap	Pat Hap
GL1	13.54%	61	24	2	9	0	13	M2	F2
GL2	7.51%	60	40	9	31	0	0	M2	NC
GL3	18.84%	75	22	0	13	9	0	M2	F1
GL4	15.39%	78	13	5	8	0	0	M2	NC
GL5	7.01%	69	19	12	2	5	0	M1	F1
GL6	18.97%	79	29	17	0	12	0	M1	F1
GL7	6.75%	55	30	14	2	0	14	M1	F2
GL8	9.48%	64	23	2	10	11	0	M2	F1
GL9	9.39%	63	23	4	9	1	9	M2	F2
GL10	8.21%	66	17	2	7	7	1	M2	F1
GL11	6.64%	58	20	12	1	0	7	M1	F2
GL12	11.01%	68	11	4	7	0	0	M2	NC
GL13	11.45%	81	22	10	3	9	0	M1	F1
GL14	9.09%	54	33	10	3	2	18	M1	F2
GL15	9.48%	67	27	5	9	11	2	M2	F1
GL16	6.21%	64	29	13	3	0	13	M1	F2
GL17	8.14%	63	27	4	12	2	9	M2	F2
GL18	10.92%	80	18	0	9	0	9	M2	F2
GL19	11.11%	71	38	21	6	0	11	M1	F2
GL20	10.00%	76	22	4	9	0	9	M2	F2
GL21	13.12%	77	29	25	4	0	0	M1	NC
GL22	6.40%	61	35	11	6	0	18	M1	F2
GL23	5.07%	30	23	9	14	0	0	M2	NC
GL24	7.02%	59	20	3	9	7	1	M2	F1
GL25	7.66%	62	15	10	5	0	0	M1	NC
GL26	5.97%	50	21	5	5	1	10	NC	F2
GL27	10.78%	72	23	1	9	1	12	M2	F2
GL28	12.06%	76	35	2	15	1	17	M2	F2
GL29	9.74%	76	22	4	9	0	9	M2	F2
GL30	7.40%	70	24	4	7	1	12	M2	F2
GL31	8.60%	71	22	7	15	0	0	M2	NC
GL32	6.17%	58	46	19	7	5	15	M1	F2
GL33	9.40%	82	27	11	5	1	10	M1	F2
GL34	11.20%	75	27	9	0	1	17	M1	F2
GL35	7.38%	57	28	4	7	7	10	M2	F2

Abbreviations: FAM: Family number; FF: Fetal fraction; Mat Hap: Maternal haplotype; Pat Hap: Paternal haplotype.

Table S5. Performance of NIPT-MD Compared with Invasive Diagnostic Testing Results

NIPT-MD Result	Gold Standard Positive (n=20)	Gold Standard Negative (n=15)	Total
Positive	20	0	20
Negative	0	15	15
Total	20	15	35

Diagnostic Performance Metric	Value % (95% CI)
Sensitivity	100.0 (83.89–100.00)
Specificity	100.0 (79.61–100.00)
PPV	100.0 (83.89–100.00)
NPV	100.0 (79.61–100.00)
Accuracy	100.0(90.11–100.00)

Abbreviations: NIPT-MD: Non-invasive prenatal diagnosis testing for monogenic disorders; CI: Confidence interval; PPV: Positive predictive value; NPV: Negative predictive value.
